# Transcriptome analysis of thymic tissues from Chinese Partridge Shank chickens with or without Newcastle disease virus LaSota vaccine injection via high-throughput RNA sequencing

**DOI:** 10.1080/21655979.2021.2008737

**Published:** 2022-04-09

**Authors:** Furong Nie, Jingfeng Zhang, Mengyun Li, Xuanniu Chang, Haitao Duan, Haoyan Li, Jia Zhou, Yudan Ji, Liangxing Guo

**Affiliations:** a Henan University of Animal Husbandry and Economy, Zhengzhou, China; b Henan Chenxia Biomedical Co., Ltd, Zhengzhou, China

**Keywords:** Newcastle disease, Newcastle disease virus, LaSota, RNA sequencing, lncRNA, microRNA, mRNA, innate immune

## Abstract

The LaSota strain of Newcastle disease virus (NDV) is a commonly used vaccine to control Newcastle disease. However, improper immunization is a common reason for vaccine failure. Hence, it is imperative to thoroughly explore innate immunity-related molecular regulatory responses to the LaSota vaccine. In this text, 140 long non-coding RNAs (lncRNAs), 8 microRNAs (miRNAs), and 1514 mRNAs were identified to be differentially expressed by RNA sequencing analysis in the thymic tissues of Chinese Partridge Shank chickens after LaSota vaccine inoculation. Moreover, 70 dysregulated genes related to innate immunity were identified based on GO, Reactome pathway, and InnateDB annotations and differential expression analysis. Additionally, dysregulated lncRNAs and innate immunity-related mRNAs that could interact with dysregulated miRNAs were identified based on bioinformatics prediction analysis via the miRanda software and differential expression analysis. Among these transcripts, expression patterns of five lncRNAs, seven miRNAs, and six mRNAs were further examined by RT-qPCR assay. Both RNA-seq and RT-qPCR outcomes showed that 10 transcripts (MSTRG.22689.1, ENSGALT00000065826, ENSGALT00000059336, ENSGALT00000060887, gga-miR-6575-5p, gga-miR-6631-5p, gga-miR-1727, paraoxonase 2 (PON2), mitogen-activated protein kinase 10, and cystic fibrosis transmembrane conductance regulator (CFTR) were highly expressed, and 4 transcripts (MSTRG.188121.10, gga-miR-6655-5p, gga-miR-6548-3p, and matrix metallopeptidase 9 (MMP9) were low expressed after NDV infection. Additionally, two potential competing endogenous RNA networks (ENSGALT00000060887/gga-miR-6575-5p/PON2 or MSTRG.188121.10/gga-miR-6631-5p/MMP9) and some co-expression axes (ENSGALT00000065826/gga-miR-6631-5p, MSTRG.188121.10/gga-miR-6575-5p, MSTRG.188121.10/CFTR, ENSGALT00000060887/MMP9) were identified based on RT-qPCR and co-expression analyses. In conclusion, we identified multiple dysregulated lncRNAs, miRNAs, and mRNAs after LaSota infection and some potential regulatory networks for these dysregulated transcripts.

## Introduction

Newcastle disease (ND), one of the leading devastating diseases in poultry and wild birds worldwide, has undergone several outbreaks and brought enormous economic losses for the poultry industry since its first official report 90 years ago (https://www.oie.int/en/animal-health-in-the-world/animal-diseases/newcastle-disease/) [1,2]. Moreover, ND is a zoonosis (an animal disease that also can infect humans) that can cause mild conjunctivitis and influenza-like symptoms in humans [2,3]. Newcastle Disease virus (NDV), a negative-sense single-stranded RNA virus, belongs to the genus of Avulavirus in the family of Paramyxoviridae [4]. NDV strains can be categorized as lentogenic (mild), mesogenic (moderate), and velogenic (very virulent) pathotypes according to the difference of mean death time in chicken embryos [5]. Lentogenic NDV strains produce mild subclinical signs with negligible mortality, while velogenic strains can cause lethal hemorrhagic lesions, serious nervous and respiratory disorders, rapid transmission, and high mortality in birds and poultry [5]. Once found, mesogenic and virulent NDV viruses need to be immediately notified to the Office of International Epizootics because virulent strains of NDV are defined as the causative agent of ND by the World Health Organization for Animals (OIE) [6]. To date, more than 250 bird species, especially gallinaceous birds (*e.g*. quail, chickens, and turkeys), have been reported with NDV infection [7,8].

Vaccination is an effective preventive strategy to protect animals from infectious diseases by stimulating the immune system [9,10]. Over the past decades, the NDV LaSota strain has been widely used as one vaccine formulation to protect poultry from virulent NDV infection and prevent ND in many countries due to its favorable immunogenicity [6,8,11]. However, vaccine failure caused by vaccines incompleteness and/or improper immunization is a common issue [11]. To better utilize vaccines and manage the vaccine failure problem, it is requisite to gain a comprehensive knowledge and understanding of molecular responses to NDV vaccines.

Coding RNAs and non-coding RNAs, including long non-coding RNAs (lncRNAs) and microRNAs (miRNAs), have emerged as crucial players in various biological processes and host responses to stress and pathogens in animals including chickens [12–14]. Recently, high-throughput RNA sequencing (RNA-Seq) and microarrays that can simultaneously capture a host of transcripts and decipher the information on transcriptome (*e.g*. lncRNAs, miRNAs, and mRNAs) have been widely used to identify differentially expressed genes under different conditions and investigate the host-pathogen interactions [15,16]. Compared to the microarray technology, RNA-seq is more accurate and sensitive with low background noise [17,18]. Moreover, RNA-seq is independent of the availability, expression intensity, and prior annotation of probes. Additionally, RNA-seq can identify novel transcripts/low abundance transcripts/biologically critical isoforms and distinguish different isoforms/allelic expression [17,18]. However, RNA-seq also faces several challenges in library construction and bioinformatic analysis [17]. For instance, to examine whether short reads that are identical to each other are genuine RNA, it is requisite to set up multiple biological replicates. To reduce errors in bioinformatic analysis, it is indispensable to remove the low-quality reads [17].

Although previous studies have performed the transcriptome analysis to explore host immune responses against NDV LaSota vaccine infection in embryos [19], spleen [20], trachea [21] from Leghorn and Fayoumi chickens and chick embryo fibroblasts [22], these documents focused on the investigation of NDV LaSota vaccine on differentially expressed genes including some innate immune genes. To our knowledge, the relationships of lncRNAs, miRNAs, and innate immunity-related mRNAs have not been examined in thymic tissue samples of Chinese Partridge Shank chickens after LaSota vaccine injection at the transcriptomic level. In this text, to gain an in-depth insight into the molecular responses to NDV LaSota vaccine in Chinese Partridge Shank chickens, differentially expressed lncRNAs, miRNAs, and mRNAs after NDV LaSota vaccine injection were identified using RNA-seq technology. Given the vital roles of improper immune responses in vaccine failure and relative conservation of innate immune responses among different species, a total of 70 dysregulated genes related to innate immunity were screened out. Moreover, some vital innate immune genes in response to NDV infection were identified by interaction analysis. To thoroughly investigate the relationships of lncRNAs, miRNAs, and mRNAs, lncRNAs or mRNAs that could interact with dysregulated miRNAs were identified by bioinformatics prediction analysis. To further decipher the lncRNA/miRNA/mRNA regulatory networks related to innate immunity, we screened out the differentially expressed lncRNAs and innate immunity-related mRNAs that could interact with dysregulated miRNAs. Among these lncRNAs, miRNAs and mRNAs, five lncRNAs, seven miRNAs, and six innate immune genes were picked out for further RT-qPCR examination in thymic tissues of 10 chickens with or without NDV infection. Based on the co-expression relationships and competing endogenous RNA (ceRNA) hypothesis, two potential ceRNA regulatory networks (MSTRG.188121.10/gga-miR-6631-5p/matrix metallopeptidase 9 (MMP9) and ENSGALT00000060887/gga-miR-6575-5p/paraoxonase 2 (PON2)) were identified.

## Materials and methods

### Sample collection

Specific pathogen-free (SPF) Chinese Partridge Shank chickens were raised in biosafety level II facilities because the NDV LaSota strain was lentogenic as previously described [23,24]. At the age of 30 days, the challenged chickens were inoculated with 0.2 ml of 10^5^50% egg-infectious dose (EID_50_) of Lasota suspension through eyes. At 0 or 48 h post-infection, chickens were euthanized, and the thymic tissue samples were collected in RNase free microtubes, immediately frozen in liquid nitrogen, and then stored at −80°C until RNA extraction. Our study was approved by the Animal Care and Use Committee of the Henan University of Animal Husbandry and Economy. All efforts were made to minimize the suffering of birds.

### Total RNA extraction, cDNA library construction, and sequencing

RNA was isolated from thymic tissue samples using Trizol Reagent (Thermo Fisher Scientific, Waltham, MA, USA) and then treated with a TURBO DNA-free Kit (Thermo Fisher Scientific) to remove DNA from RNA samples (Thermo Fisher Scientific) as previously described [25]. RNA sequencing (RNA-seq) samples were prepared, and cDNA libraries were constructed and sequenced as previously described [26–28]. Briefly, the concentration and purity of RNA were measured using NanoDrop 2000 spectrophotometer (Thermo Fisher Scientific), and RNA quality and integrity were further tested using an RNA Nano 6000 Assay Kit (Agilent Technologies, Santa Clara, CA, USA) through Agilent Bioanalyzer 2100 (Agilent Technologies). After the removal of ribosomal RNA using the Ribo-Zero rRNA Removal Kit (Illumina), high-quality samples (RNA concentration ≥400 ng/μl, OD260/280: 1.8–2.2, RNA Integrity number ≥8) and the TruSeq Stranded Total RNA Library Prep Kit (Illumina, San Diego, CA, USA) were used for the construction of cDNA libraries of mRNAs and lncRNAs according to the instructions of the manufacturer. For the construction of cDNA libraries of RNAs containing lncRNAs and mRNAs, the remaining RNAs were purified and fragmented. Next, fragmented mRNAs were reversely transcribed into cDNAs, followed by the conversion of residual overhangs into blunt ends. Subsequently, Illumina PE adapter oligonucleotides were ligated with adenylated DNAs (3’ends). Subsequently, cDNA fragments were enriched by PCR amplification and purified through the Agencourt AMPure XP system (Beckman Coulter, Beverly, CA, USA). Next, cDNA libraries were quantified by the Agilent high-sensitivity DNA assay on Agilent Bioanalyzer 2100 (Agilent Technologies). Finally, single-strand cDNA libraries were sequenced on Illumina HiSeq 2500 instrument (Illumina) by Shanghai Personal Biotechnology Co. Ltd. (Shanghai, China). Small RNA libraries were conducted through NEBNext Multiplex Small RNA Library Prep Set for Illumina (New England Biolabs, Ipswich, MA, USA) following the instructions of the manufacturer. Briefly, RNA samples were ligated to RNA 3’ and 5’ adapters and then reverse-transcribed and amplified into cDNA libraries. After being analyzed by Agilent Bioanalyzer 2100 (Agilent Technologies), libraries were sequenced. The sequence data have been uploaded to the NCBI with the accession number PRJNA600558.

### Raw data processing

The information of the reference genome (Gallus_gallus.Gallus_gallus-5.0 (assembly GCA_000002315.3) is shown in Table 1. Annotation information of Gallus_gallus.Gallus_gallus-5.0 (assembly GCA_000002315.3) genes in different databases was displayed in Table 2. Information on the raw data is presented in Table 3. After filtering, high-quality clean data without reads containing 3’ adaptors or average base quality < Q20 were aligned to the reference genome (Gallus_gallus.Gallus_gallus-5.0 (assembly GCA_000002315.3)) using Tophat2 as previously described [26]. The alignment results are presented in Table 4.

**Table 1.** Reference genome information

Genome	Gallus_gallus.Gallus_gallus-5.0.dna.toplevel.fa
genebuild by	Ensembl
Database version	94.5
Base Pairs	1,285,637,921

**Table 2.** Chicken gene annotation information in different databases

Database	Number	Percentage
NCBI_GI	12312	67.1
eggNOG	1158	6.31
KEGG	9014	49.13
GO	15747	85.83
EC	2675	14.58
UniProtID	8761	47.75
Ensembl	18,346	100

**Table 3.** Raw data statistics

Sample	Reads No.	Bases (bp)	Q30 (bp)	N (%)	Q20 (%)	Q30 (%)
ctl1	112,006,994	16,913,056,094	15,556,184,034	0.016238	96.64	91.97
ctl2	107,861,238	16,287,046,938	14,921,050,839	0.010633	96.46	91.61
ctl3	101,546,874	15,333,577,974	14,003,424,760	0.005531	96.26	91.32
48h1	108,620,036	16,401,625,436	15,386,850,552	0.002824	97.37	93.81
48h2	106,833,502	16,131,858,802	15,119,130,773	0.002845	97.34	93.72
48h3	101,846,738	15,378,857,438	14,396,829,696	0.002809	97.26	93.61

**Note:**

Bases (bp): Total number of bases.

Q30 (bp): Base number of base recognition accuracy rate > 99.9%.

N (%): Percentage of ambiguous bases.

Q20 (%): Percentage of bases with recognition accuracy rate > 99%.

Q20 (%): Percentage of bases with recognition accuracy rate > 99.9%.

**Table 4.** RNAseq Map statistics

Sample	Clean_Reads	Total_Mapped	Multiple_Mapped	Uniquely_Mapped
ctl1	111,188,706	90,835,667 (81.70%)	3,410,734 (3.75%)	87,424,933 (96.25%)
ctl2	106,983,056	87,490,661 (81.78%)	3,258,412 (3.72%)	84,232,249 (96.28%)
ctl3	100,626,522	80,628,531 (80.13%)	3,009,815 (3.73%)	77,618,716 (96.27%)
48h1	108,187,642	88,886,144 (82.16%)	3,497,429 (3.93%)	85,388,715 (96.07%)
48h2	106,428,002	87,593,246 (82.30%)	3,605,116 (4.12%)	83,988,130 (95.88%)
48h3	101,429,340	82,519,425 (81.36%)	3,459,058 (4.19%)	79,060,367 (95.81%)

**Note:**

Clean Reads No.: total number of aligned sequences

Total Mapped: sequence number aligned to reference genome, percentage: Total Mapped number/Clean Read number

Multiple Mapped: sequence number aligned to multiple positions, percentage: Multiple Mapped number/Total Mapped number

Uniquely Mapped: sequence number aligned to one position, percentage: Uniquely Mapped number/Total Mapped number

### Differential expression analysis

Expression levels of lncRNAs and mRNAs were measured using the fragments per kilobase of transcript per million mapped reads (FPKM) method as previously described [26]. Difference expression analysis was performed using the DESeq R package. Differentially expressed genes were screened out using the conditions of |log_2_FoldChange| >1 and *P-*value <0.05. The MA plot was drawn using the ggplot2 R package.

### Functional annotation and enrichment analysis of differentially expressed genes

Genes were annotated using the Gene Ontology (GO) (http://geneontology.org/), Kyoto Encyclopedia of Genes and Genomes (KEGG) (http://www.kegg.jp/), UniProt Knowledgebase (http://www.uniprot.org/help/uniprotkb/), Enzyme Commission (EC) (http://enzyme.expasy.org/), Evolutionary Genealogy of Genes: Non-supervised Orthologous Groups (eggNOG) (http://eggnog.embl.de/version_3.0/) databases. miRNA annotation analysis was performed using the miRbase database. GO terms and KEGG pathways were regarded to be significantly enriched when the *P*-value was less than 0.05 [29].

### Target prediction and venn analysis

The potential interactions between miRNAs and lncRNAs or mRNAs were predicted by the miRanda software. Venn analysis was performed using the jvenn website (http://jvenn.toulouse.inra.fr/app/index.html).

### Reverse transcription-quantitative PCR (RT‐qPCR)

RNA was extracted using Trizol Reagent (Thermo Fisher Scientific) and quantified using NanoDrop 2000 spectrophotometer (Thermo Fisher Scientific) as described above. Next, the reverse transcription and quantitative PCR reactions were performed as previously described [30,31]. Briefly, RNA was reversely transcribed into cDNA first strands using an iScript cDNA Synthesis Kit (Bio-Rad, Hercules, CA, USA) and random primer (for mRNAs and lncRNAs) or stem-loop miRNA RT primers in Table 5. Next, cDNA was amplified using ITaq Universal SYBR Green Supermix (Bio-Rad) and specific primers in Table 6. Expression levels of lncRNAs, mRNAs, and miRNAs were calculated using the formula of 2^−ΔΔCT^ with GAPDH or 5S rRNA as the internal control [32,33].

**Table 5.** Reverse-transcription primers for miRNAs (stem-loop primers)

gga-miR-6631-5p	5’-CTCAACTGGTGTCGTGGAGTCGGCAATTCAGTTGAGgcagaacc-3’
gga-miR-6655-5p	5’-CTCAACTGGTGTCGTGGAGTCGGCAATTCAGTTGAGaggataca-3’
gga-miR-6575-5p	5’-CTCAACTGGTGTCGTGGAGTCGGCAATTCAGTTGAGaagagctt-3’
gga-miR-6548-3p	5’-CTCAACTGGTGTCGTGGAGTCGGCAATTCAGTTGAGacagcaac-3’
gga-miR-1744-3p	5’-CTCAACTGGTGTCGTGGAGTCGGCAATTCAGTTGAGtcagtctt-3’
gga-miR-1727	5’-CTCAACTGGTGTCGTGGAGTCGGCAATTCAGTTGAGccttcagt-3’
gga-miR-1712-3p	5’-CTCAACTGGTGTCGTGGAGTCGGCAATTCAGTTGAGccaaactc-3’

**Table 6.** Quantitative PCR primers

miRNAs	Forward primers	Reverse primers
gga-miR-6631-5p	5’-ACACTCCAGCTGGGGAAGAGAATGCTGTGG-3’	5’-TGGTGTCGTGGAGTCG-3’
gga-miR-6655-5p	5’-ACACTCCAGCTGGGTCTGCTAGGAGGCTGTG-3’	5’-TGGTGTCGTGGAGTCG-3’
gga-miR-6575-5p	5’-ACACTCCAGCTGGGTTGTCAGCTTGGGGAA-3’	5’-TGGTGTCGTGGAGTCG-3’
gga-miR-6548-3p	5’-ACACTCCAGCTGGGAGAGGTGCCCCGCTGT-3’	5’-TGGTGTCGTGGAGTCG-3’
gga-miR-1744-3p	5’-ACACTCCAGCTGGGACTTCAACAGGAGCAA-3’	5’-TGGTGTCGTGGAGTCG-3’
gga-miR-1727	5’-ACACTCCAGCTGGGAAGCTGCTCTAATGAAC-3’	5’-TGGTGTCGTGGAGTCG-3’
gga-miR-1712-3p	5’-ACACTCCAGCTGGGTTCAGTTATCAGTGGA-3’	5’-TGGTGTCGTGGAGTCG-3’
mRNAs	forward primers	reverse primers
ENSGALG00000009689 (PON2)	5’-CCTCATGGGATCAGCACTTAC-3’	5’-CACTTGTCAGAAGGTCGTGTC-3’
ENSGALG00000003045 (E2F1)	5’-CACCACAGCCACAGGATTAC-3’	5’-GAAGTCCCCAAAGTCACAGTC-3’
ENSGALG00000032986 (CFTR)	5’-GGGGAGGTCACCAAATCTGT-3’	5’-AGGGTGTATGAGCAGCGTTC-3’
ENSGALG00000011109 (MAPK10)	5’-TACAGCGTGGAAGTGGGTGA-3’	5’-GCACAAACTATTCCCTGAGCC-3’
ENSGALG00000006992 (MMP9)	5’-CCACTCGTCCTTCTGGAAATC-3’	5’-CCCTCTTGGTGAGCACATCTT-3’
ENSGALG00000005653 (NF-κB2)	5’-AGCAGTGGGAACTTCACTCTG-3’	5’-TCGTCGTCCTCATAGAACCG-3’
lncRNAs	forward primers	reverse primers
MSTRG.188121.10	5’-AGACGTTACAGCAACCTGGG-3’	5’-AGTCCTTGGACTGAGCAACG-3’
ENSGALT00000065826	5’-GACAGGACGTTTGCTCCTCA-3’	5’-CTTCTCCCCCACATAACGCA-3’
ENSGALT00000059336	5’-GGAGAAGACGGGCATTTCCT-3’	5’-TTAGACCGTTTTGCCCCCAG-3’
ENSGALT00000060887	5’-CGGAAAGTGTCGTTGCAGTC-3’	5’-AGAGCAGACGCATCTGAAGG-3’
MSTRG.22689.1	5’-GTTCCAGGCATCTCGGGTTT-3’	5’-AATCAGGCTTGCCACCATACT-3’

### Statistical analysis

Data were analyzed using GraphPad Prism 7 software (GraphPad Software, Inc., San Diego, CA, USA). The student’s *T-*test was used to compare the difference between groups. The difference was regarded to be statistically significant when the *P*-value was less than 0.05.

## Results

In this project, 140 lncRNAs, 8 miRNAs, and 1514 mRNAs were identified to be differentially expressed in thymic tissues of chickens after NDV vaccine inoculation. Improper immunization is a common cause of vaccine failure. Thus, we believed that the dysregulation of innate immune genes was closely linked with vaccine failure. In this project, 70 differentially expressed innate immune genes were screened out based on differential expression analysis, GO enrichment analysis, and annotation information in the InnateDB and Reactome Pathway databases. To identify key innate immune genes, the interaction networks of these dysregulated innate immune genes were established, and the number of neighboring genes of each node in the network was calculated. We supposed that genes with more neighboring genes might play vital roles in the innate immune responses to NDV infection. Moreover, the major pathways and biological processes that were significantly enriched by these dysregulated innate immune genes were identified by KEGG and GO enrichment analysis. To decipher the regulatory relationships of these dysregulated lncRNAs, miRNAs, and mRNAs, lncRNAs and mRNAs that could interact with differentially expressed miRNAs were predicted by the miRanda software. Moreover, differentially expressed lncRNAs and innate immune genes that could bind with dysregulated miRNAs were identified. Additionally, the expression patterns of five lncRNAs, seven miRNAs, and six innate immune genes were further examined by RT-qPCR assay in thymic tissues of 10 chickens with or without NDV infection. Furthermore, the co-expression relationships of these lncRNAs, miRNAs, and mRNAs were investigated by correlation analysis. The results showed that gga-miR-6631-5p expression was negatively associated with MSTRG.188121.10 or MMP9 expression, and MSTRG.188121.10 expression was positively correlated with MMP9 expression. Also, there was a negative regulatory relationship between gga-miR-6575-5p and ENSGALT00000060887 or PON2, and there was a positive correlation between ENSGALT00000060887 and PON2. A large body of evidence shows that lncRNAs can act as the molecular sponge of miRNAs to alleviate the inhibitory effects of miRNAs on target genes. Hence, we supposed that MSTRG.188121.10 might function as a molecular sponge of gga-miR-6631-5p to regulate MMP9 expression, and ENSGALT00000060887 might regulate PON2 expression by sequestering gga-miR-6575-5p.

## Identification of differentially expressed lncRNAs

**Figure 1.**
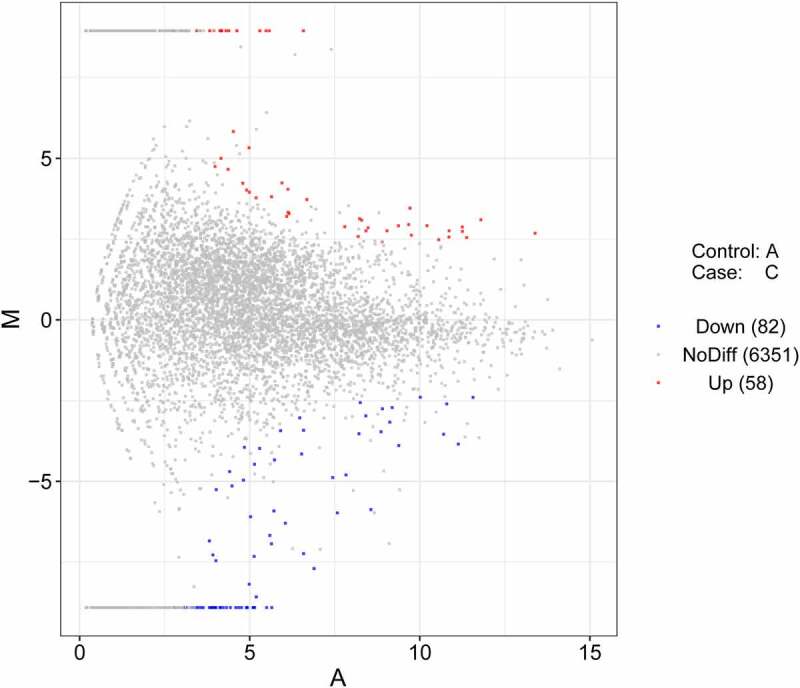
The MA plots of differentially expressed lncRNAs in the vaccine-treated group vs the control group.

RNA-seq results showed that 140 lncRNAs were differentially expressed (|log_2_FoldChange| >1, *P*-value <0.05) in thymic tissue samples of chickens after NDV vaccine treatment versus the control group (Supplementary Table 1, Figure 1).

## Identification of differentially expressed miRNAs and potential miRNA targets

Eight miRNAs (3 up-regulated, 5 down-regulated) (|log_2_FoldChange| >1, *P*-value <0.05) were found to be differentially expressed in thymic tissues of chickens after vaccine treatment compared to the control group (Data have been shown in Supplementary Table 5 (C vs A group) in our recently published article [34]). In detail, expression levels of gga-miR-6631-5p, gga-miR-6575-5p, and gga-miR-1727 were noticeably increased and expression levels of gga-miR-1712-3p, gga-miR-12273-3p, gga-miR-6655-5p, gga-miR-1744-3p, and gga-miR-6548-3p were markedly reduced in thymic tissue samples of chickens in response to NDV vaccine injection. It is well known that miRNAs can exert their functions by regulating specific target mRNAs. Hence, potential targets of differentially expressed miRNAs were identified by bioinformatics analysis using the miRanda software, and the results were presented in Supplementary Table 2.

## Identification of differentially expressed mRNAs and related KEGG enrichment analysis

Moreover, 1016 up-regulated mRNAs and 498 down-regulated mRNAs (|log_2_FoldChange| >1, *P*-value <0.05) were identified in thymic tissue samples of chickens at 48 h after NDV vaccine infection compared to the control group (Data have been shown in Supplementary Table 7 in our recently published article [34]). KEGG enrichment analysis revealed that these dysregulated genes in response to NDV vaccine infection might be mainly involved in the regulation of immune system-related pathways, such as systemic lupus erythematosus, *Staphylococcus aureus* infection, complement and coagulation cascades, and asthma (Data have been shown in Supplementary Table 11 (C vs A group) in our recently published article [34]).

## Identification of vaccine-influenced innate immune genes

Furthermore, 17 differentially expressed genes were identified to be significantly enriched in biological processes related to innate immunity, which was screened out through searching for the GO terms including the keyword of innate immune in GO enrichment analysis of the dysregulated genes (GO enrichment analytical results have been shown in Supplementary Table 13 sheet 1 in our recently published article [34]). The information of these genes was shown in Supplementary Table 3. Given the relative conservation of innate immune responses among diverse organisms, 712 (Data have been shown in Supplementary Table 17 in our recently published article [34]), and 319 (Data have been shown in Supplementary Table 19 in our recently published article [34]) innate immune genes were identified from the InnateDB (http://innatedb.sahmri.com/annotatedGenes.do?type=innatedb,) and Reactome Pathway databases (http://reactome.ncpsb.org/PathwayBrowser/#/R-GGA-168249&PATH=R-GGA-168256&DTAB=MT), respectively. Combined with the differential expression data, 50 innate immune genes that were annotated by the InnateDB database (Data have been shown in Supplementary Table 18 sheet 3 in our recently published article [34]) and 15 innate immune genes that were annotated by the Reactome Pathway database (Data have been shown in Supplementary Table 20 sheet 3 in our recently published article [34]) were found to be differentially expressed in the post-inoculation group versus the control group. Taken together, a total of 70 differentially expressed genes related to innate immunity were screened out (Supplementary 4).

## Establishment of interaction networks of proteins encoded by these filtered innate immune genes

**Figure 2.**
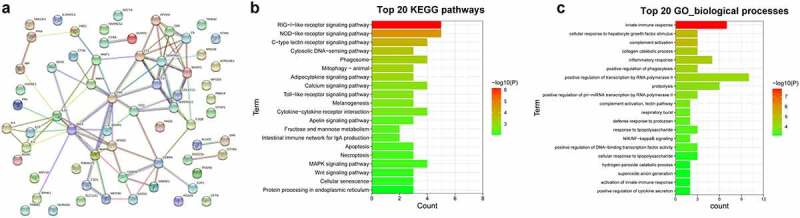
**Interaction and enrichment analysis of filtered dysregulated innate immune genes**. (a) Interaction network of proteins encoded by filtered dysregulated innate immune genes. The confidence score was equal to or higher than 0.4. (b) The top 20 KEGG pathways enriched by the dysregulated innate immune genes. (c) The top 20 GO_biological_process terms enriched by the dysregulated innate immune genes.

Next, the interaction relationships of proteins that were encoded by the above-dysregulated innate immune genes were analyzed through STRING: functional protein association networks (https://string-db.org/cgi/network?taskId=blvCWmQYCH9w&sessionId=bzHnhukrXlVB). The image of interaction networks is shown in Figure 2(a). The information of these proteins with an interaction score ≥0.4 was presented in Supplementary Table 5. The number of neighboring genes of each node was also examined, and the results were shown in sheet 3 of Supplementary Table 5. Results showed that some genes (*e.g*. mitogen-activated protein kinase 10 (MAPK10), nuclear factor kappa B subunit 2 (NF-κB2), MMP9) had more neighboring genes, suggesting that these genes might play vital roles in the interaction networks and NDV-related innate immune responses. Moreover, KEGG enrichment analysis revealed that these 70 dysregulated innate immune genes were significantly enriched in retinoic acid‑inducible gene I (RIG‑I)-like receptor (RLR), NOD-like receptor (NLR), C-type lectin receptor (CLR), Toll-like receptor (TLR), and mitogen-activated protein kinase (MAPK) signaling pathways (Supplementary Table 6 sheet 1, Figure 2(b)). As shown in Supplementary Table 6 sheet 1, MAPK10 functioned as crucial roles in RLR, NLR, CLR, TLR, and MAPK signaling pathways and NF-κB2 participated in the regulation of CLR and MAPK signaling pathways. In addition, GO enrichment analysis of these 70 dysregulated innate immune genes showed that these genes mainly participated in the regulation of biological processes, such as complement activation, inflammatory response, phagocytosis, and NIK/NF-kappaB signaling, cycle (Supplementary Table 6 sheet 2). The top 20 KEGG pathways and GO_biological_process terms that were significantly enriched by the 70 dysregulated innate immune genes are shown in Figure 2(b,c), respectively.

## Potential interaction networks of dysregulated lncRNAs, miRNAs, and innate immune genes

**Figure 3.**
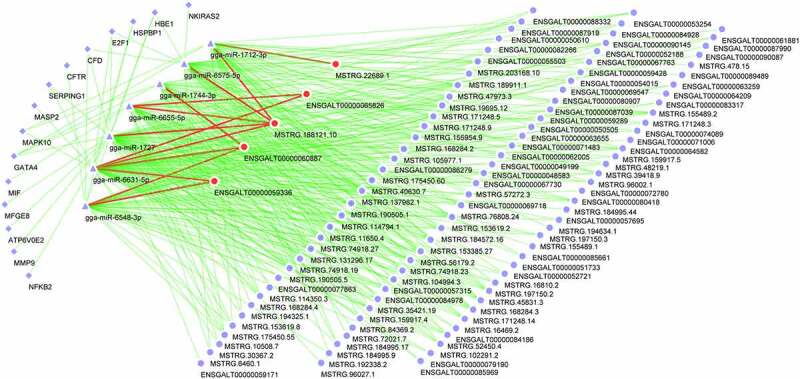
The network of differentially expressed lncRNAs, miRNAs and innate immune genes. The networks were drawn using the Cytoscape software.

Moreover, lncRNAs and mRNAs that could interact with miRNAs were predicted by the miRanda software. Based on the prediction outcomes and differential expression data, we screened out differentially expressed lncRNAs that could interact with dysregulated miRNAs. The results were shown in Supplementary Table 7. No dysregulated lncRNAs that could bind to gga-miR-12273-3p were identified. Moreover, combined with the data of putative targets of miRNAs in Supplementary Table 2 and differentially expressed genes, potential dysregulated targets of gga-miR-6631-5p, gga-miR-6575-5p, and gga-miR-1727, gga-miR-1712-3p, gga-miR-12273-3p, gga-miR-6655-5p, gga-miR-1744-3p, or gga-miR-6548-3p were screened out by jvenn analysis and shown in Supplementary Table 8. Additionally, the dysregulated innate immunity-related targets of gga-miR-6575-5p, gga-miR-6631-5p, gga-miR-1727, gga-miR-6655-5p, gga-miR-6548-3p, gga-miR-1744-3p, and gga-miR-1712-3p were identified by jvenn analysis of corresponding miRNA targets in Supplementary Table 2 and filtered dysregulated innate immune genes, and the results were shown in Supplementary Table 9 and Figure (3). No common genes were found by jvenn analysis between putative gga-miR-12273-3p target group and filtered dysregulated innate immune gene group.

## RT-qPCR validation of some differentially expressed lncRNAs, miRNAs, and mRNAs

**Figure 4.**
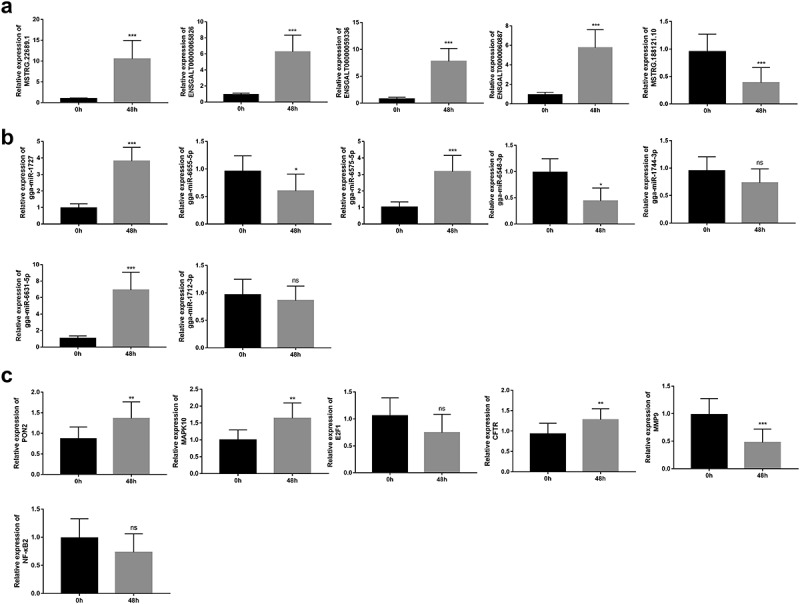
RT-qPCR validation of some differentially expressed lncRNAs, miRNAs and mRNAs. (a-c) Expression levels of several lncRNAs (MSTRG.22689.1, ENSGALT00000065826, ENSGALT00000059336, ENSGALT00000060887 and MSTRG.188121.10) (a), miRNAs (gga-miR-1744-3p, gga-miR-1712-3p gga-miR-6575-5p, gga-miR-6631-5p, gga-miR-1727, gga-miR-6655-5p and gga-miR-6548-3p) (b) and mRNAs (PON2, MAPK10, CFTR, MMP9, E2F1, and NF-κB2) (c) were further examined through RT-qPCR assay in thymic tissue samples of 10 random chickens at 0 h and 48 h post NDV injection. Note: paraoxonase 2 (PON2), mitogen-activated protein kinase 10 (MAPK10), E2F transcription factor 1 (E2F1), cystic fibrosis transmembrane conductance regulator (CFTR), matrix metallopeptidase 9 (MMP9), nuclear factor kappa B subunit 2 (NF-κB2).

Next, five lncRNAs (MSTRG.22689.1, ENSGALT00000065826, ENSGALT00000059336, ENSGALT00000060887, and MSTRG.188121.10) with higher basemean values were selected for further RT-qPCR detection. Results showed that the expression levels of MSTRG.22689.1, ENSGALT00000065826, ENSGALT00000059336, and ENSGALT00000060887 were notably up-regulated, and MSTRG.188121.10 expression level was markedly down-regulated in thymic tissue samples of chickens at 48 h upon NDV challenge compared to the control group (Figure 4(a)). Also, our data revealed that gga-miR-1727, gga-miR-6575-5p, and gga-miR-6631-5p were highly expressed, while gga-miR-6655-5p and gga-miR-6548-3p were low expressed in thymic tissue samples of chickens after NDV vaccine inoculation (Figure 4(b)). However, there was no conspicuous difference in the expression levels of gga-miR-1744-3p and gga-miR-1712-3p between the control group and the post-inoculation group (Figure 4(b)). Among the filtered genes in Supplementary Table 9, MAPK10 and cystic fibrosis transmembrane conductance regulator (CFTR), E2F transcription factor 1 (E2F1), NF-κB2, and MMP9 were screened out for further RT-qPCR exploration. Also, the expression alteration of an interested gene PON2 in response to NDV infection was examined by RT-qPCR assay. Results showed that PON2, MAPK10, and CFTR were highly expressed, and MMP9 were low expressed in the thymic tissue samples of chickens after NDV vaccine injection relative to the control group (Figure 4(c)). However, there was no obvious alteration in the expression levels of E2F1 and NF-κB2 after NDV vaccine inoculation in the thymic tissues of chickens (Figure 4(c)).

## Co-expression analysis of dysregulated lncRNAs, miRNAs, and mRNAs

**Figure 5.**
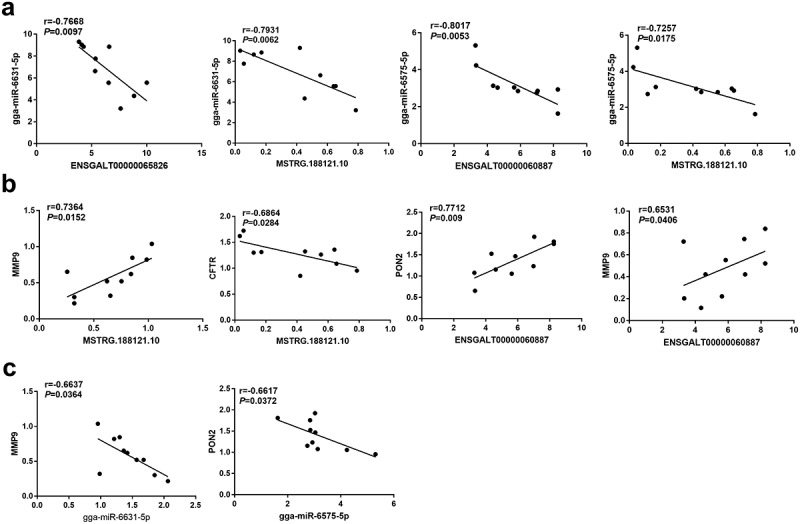
Pearson correlation analysis of differentially expressed lncRNAs, miRNAs and mRNAs in thymic tissue samples of 10 random chickens at 48 h after NDV inoculation. Note: matrix metallopeptidase 9 (MMP9), cystic fibrosis transmembrane conductance regulator (CFTR), paraoxonase 2 (PON2).

Next, Pearson correlation analysis revealed that gga-miR-6631-5p expression level was negatively associated with the expression level of ENSGALT00000065826 or MSTRG.188121.10 in thymic tissue samples of chickens after NDV vaccine injection (Figure 5(a)). Moreover, there was a negative correlation between gga-miR-6575-5p expression and ENSGALT00000060887 or MSTRG.188121.10 expression in thymic tissue samples of chickens after NDV vaccine challenge (Figure 5(a)). In addition, MSTRG.188121.10 expression was positively related to MMP9 expression but was inversely correlated with CFTR expression in thymic tissue samples of chickens following NDV vaccine treatment (Figure 5(b)). ENSGALT00000060887 expression was positively associated with PON2 or MMP9 expression in thymic tissue samples of chickens after NDV vaccine administration (Figure 5(b)). Moreover, there was a negative correlation between gga-miR-6631-5p and MMP9 expression in thymic tissue samples of chickens after NDV vaccine administration (Figure 5(c)). Additionally, gga-miR-6575-5p expression was inversely correlated with PON2 expression in thymic tissue samples of chickens after NDV vaccine challenge (Figure 5(c)). Based on the co-expression data of ENSGALT00000060887, gga-miR-6575-5p, and PON2, we supposed that ENSGALT00000060887 might function as a molecular sponge of gga-miR-6575-5p to sequester gga-miR-6575-5p from PON2 in thymic tissues of chickens after NDV vaccine injection. MSTRG.188121.10 might regulate MMP9 expression through acting as a ceRNA of gga-miR-6631-5p in thymic tissues of chickens after NDV vaccine injection.

## Discussion

In this text, expression levels of 58 lncRNAs, 3 miRNAs, and 1016 mRNAs were identified to be notably up-regulated, and expression levels of 82 lncRNAs, 5 miRNAs, and 498 mRNAs were found to be markedly down-regulated in thymic tissues of Chinese Partridge Shank chickens after LaSota NDV vaccine inoculation compared with the control group without vaccine injection.

Live vaccines are known for their stronger protective effects due to their capacity to efficiently induce a series of robust immune responses [6,11,35]. Given the conservation of innate immune responses among different organisms [36], plenty of innate immune genes were identified through GO, InnateDB and Reactome Pathway analysis. Among these innate immune genes, we found that 70 innate immune genes (31 up-regulated, 39 down-regulated) were differentially expressed in the vaccine treatment group versus the control group. KEGG enrichment analysis of the dysregulated innate immune genes disclosed that these genes were involved in the regulation of RLR, NLR, CLR, TLR, and MAPK signaling pathways, while these pathways have been identified as crucial players in innate immune responses to pathogens [37–40]. For instance, the activation of TLR4 and TLR3 pathways could enhance the immune responses to the NDV vaccine (R2B-mesogenic strain) in chicken [41]. RIG-I overexpression could protect cells from NDV infection [42]. KEGG enrichment analysis also showed that MAPK10 participated in the regulation of RLR, NLR, CLR, TLR4, and MAPK signaling pathways and NF-κB2 were involved in CLR and MAPK signaling pathways. Moreover, the protein–protein interaction analysis of these dysregulated innate immune genes with known names showed that MAPK10, MMP9, and NF-κB2 might be crucial players in the innate immune responses to NDV infection. MAPK10 [43], MMP9 [44,45] and NF-κB2 [46,47] also have been reported to be involved in the regulation of innate immune responses. These data suggested the vital roles of MAPK10, MMP9, and NF-κB2 in the immune responses to NDV infection. In this project, we demonstrated that MAPK10 was highly expressed and MMP9 was low expressed in thymic tissue samples of chickens after NDV vaccine challenge. However, RT-qPCR assay showed that there was no obvious difference in NF-κB2 expression between the NDV treatment group and the uninfected group. Additionally, both RNA-seq and RT-qPCR outcomes demonstrated that PON2 and CFTR expression levels were notably increased in response to NDV vaccine infection in thymic tissues of chickens. PON2, a member of the paraoxonase (PON) family, has been found to be implicated in innate immune responses [48]. For instance, Devarajan et al. demonstrated that PON2 loss hindered bacterial clearance and facilitated macrophage phagocytosis in mice infected with *Pseudomonas aeruginosa* [49]. CFTR also have been identified to be vital players in innate immune responses [50,51].

Additionally, both RNA-seq and RT-qPCR outcomes showed that expression levels of seven transcripts (*i.e*. MSTRG.22689.1, ENSGALT00000065826, ENSGALT00000059336, ENSGALT00000060887, gga-miR-6575-5p, gga-miR-6631-5p, and gga-miR-1727) were notably increased, and expression levels of 3 transcripts (*i.e*. MSTRG.188121.10, gga-miR-6655-5p, and gga-miR-6548-3p) were markedly reduced in thymic tissue samples of chickens after NDV challenge relative to the control group. Furthermore, we demonstrated that there was a negative association between gga-miR-6631-5p and ENSGALT00000065826/MSTRG.188121.10/MMP9, between gga-miR-6575-5p and ENSGALT00000060887/MSTRG.188121.10/PON2, between MSTRG.188121.10 and CFTR in thymic tissue samples of 10 chickens after NDV vaccine injection. ENSGALT00000060887 expression was positively correlated with the expression of PON2 or MMP9 in thymic tissue samples of 10 chickens after NDV vaccine challenge. MSTRG.188121.10 expression was positively associated with MMP9 expression in thymic tissue samples of 10 chickens after NDV vaccine inoculation. Recently, the ceRNA hypothesis has attracted much attention from researchers in elucidating the molecular basis of non-coding RNAs including lncRNAs [52]. The ceRNA hypothesis proposes that lncRNAs can perform as the molecular sponge of miRNAs to attenuate the inhibitory effect of miRNAs on target genes [53–55]. Based on the co-expression analysis, we supposed that MSTRG.188121.10 might act as a ceRNA of gga-miR-6631-5p to regulate MMP9 expression, and ENSGALT00000060887 might regulate PON2 expression by functioning as a molecular sponge of gga-miR-6575-5p in chickens after NDV vaccine inoculation.

## Conclusions

In this study, 140 lncRNAs, 8 miRNAs, and 1514 mRNAs were identified to be differentially expressed in thymic tissues of Chinese Partridge Shank chickens after LaSota NDV vaccine inoculation. Moreover, 70 dysregulated innate immune genes after LaSota injection were identified. Furthermore, dysregulated lncRNAs and innate immune genes that could interact with dysregulated miRNAs were filtered out. Additionally, two potential ceRNA networks (ENSGALT00000060887/gga-miR-6575-5p/PON2 and MSTRG.188121.10/gga-miR-6631-5p/MMP9) and multiple possible lncRNA/miRNA and miRNA/mRNA regulatory axes were identified for the first time. These data could deepen our understanding of molecular responses to LaSota infection and contribute to better management of vaccine failure problems.

## Highlights

1. NDV infection triggered the dysregulation of 140 lncRNAs, 8 miRNAs, and 1514 mRNAs.

2. A total of 70 innate immune genes were dysregulated after NDV infection.

3. Ten co-expression axes of dysregulated lncRNAs, miRNAs, and mRNAs were identified.

4. MSTRG.188121.10 might regulate MMP9 expression by sponging gga-miR-6631-5p.

5. ENSGALT00000060887 might regulate PON2 expression by sequestering gga-miR-6575-5p.

## Supplementary Material

Supplemental Material

## Data Availability

The data displayed in this manuscript are available from the corresponding author upon reasonable request.
